# Overlapping exposure effects of pathogen and dimethoate on honeybee (*Apis mellifera* Linnaeus) metabolic rate and longevity

**DOI:** 10.3389/fphys.2023.1198070

**Published:** 2023-06-06

**Authors:** Kaarel Pent, Sigmar Naudi, Risto Raimets, Margret Jürison, Egle Liiskmann, Reet Karise

**Affiliations:** Estonian University of Life Sciences, Tartu, Estonia

**Keywords:** owerlapping exposure, nosemosis, dimethoate (PubChem CID, 3082), metabolic rate, longevity, honeybee

## Abstract

**Introduction:** Declines in honeybee abundance have been observed worldwide during last decades. This is partly due to plant protection agents used in intensive farming, landscaping and infrastructure maintenance. Another type of factors negatively affecting honeybees is the spread of diseases caused by different pathogens and pests. Lately, more focus has been paid to the interactions between different overlapping stressors affecting honeybee health, the combination of these often being more detrimental compared to individual stressors. The most widely used stress-evaluating methods take into account lethal- or motorial changes of the individuals or colonies. Comparatively little honeybee research has examined changes in initial recovery potential and physiological symptoms of toxification. The aim of this study was to examine the combined effect of *Nosema apis* and *N. ceranae* (according to a newer classification *Vairimorpha apis* and *V. ceranae*), the common causes of nosemosis in the honeybee *Apis mellifera* L., with the insecticide dimethoate.

**Methods:** In this study, honeybee mortality and metabolic rate were used to assess the combined effects interactions of *Nosema* ssp. and dimethoate.

**Results:** Our results showed that exposure to the low concentration of either dimethoate, either one or both species of *Nosema* ssp as single factors or in the combination had no significant effect on honeybee metabolic rate. The mortality increased with the two *Nosema* spp., as well as with infection by *N. ceranae* alone. The effect of dimethoate was observed only in combination with *N. apis* infection, which alone had no effect on individual honeybee mortality.

**Conclusion: **This study demonstrates that the overlapping exposure to a non-lethal concentration of a pesticide and a pathogen can be hidden by stronger stressor but become observable with milder stressors.

## Introduction

Over the past half century, a number of problems have emerged due to agriculture and poor nature conservation practices, to which little attention has been paid. Stressors like disease-causing pathogens and parasites, the use of plant protection products, climate change and their interactions are considered as factors affecting pollinator populations (Hristov et al., 2020; Insolia et al., 2022; Piot et al., 2022). Chemical pesticides used in intensive agriculture often get the most attention in this regard. The consistent and sometimes excessive use of these has exacerbated the problem of pesticide residues accumulating in the environment (Sharma et al., 2019), where they come into contact with non-target organisms like pollinators (Shafeeque, Ahmad, and Kamal, n.d.; Nicholls and Altieri, 2013). Traces of chemical pesticides used in agriculture have been detected in various beekeeping products as well as in bees themselves (Kasiotis et al., 2014; Kiljanek et al., 2017; Böhme et al., 2018). The bee declines have been observed in America, Europe, and many other parts of the world, especially in areas where intensive farming practices are used (Dirzo et al., 2014; Potts et al., 2010; Potts et al., 2016; van Engelsdorp and Meixner, 2010; Osterman et al., 2021).

The majority of honeybee colony losses occur during the winter, but it is difficult to pinpoint the specific cause of the mortality, and it is likely the case that poor bee health often results not from single stressors, but rather combinations of stressors to which bees are exposed (Engelsdorp et al., 2009; Goulson et al., 2015; Insolia et al., 2022; van). While the parasitic varroa mite *Varroa destructor* is considered the most harmful pest to honeybees, partly because of vectoring other viruses (EPILOBEE Consortium et al., 2016), there are also other diseases which should not remain understudied. The two microsporidian pathogens, *Nosema apis* and *N. ceranae* (according Tokarev et al. (2020) *Vairimorpha apis* and *V. ceranae*) cause nosemosis, a serious but mostly not deadly disease (Botías et al., 2013). *Nosema ceranae*, originally a parasite of the Asian honeybee which nowadays causes serious problems to European honeybees all over the world and has largely replaced the *Nosema apis*, an original parasite of European honeybee (Paxton et al., 2007). Both pathogenic species infect the epithelical cells of the honeybee midgut, causing energetic stress (Mayack and Naug 2009; Zheng et al., 2014). Honeybee nosemosis is a disease that often has no clear clinical symptoms and there is no specific treatment against it (Galajda et al., 2021). Partly because of this, nosemosis is considered to be among the factors seriously affecting the overwintering success of honeybees (Fries, Ekbohm, and Villumstad, 1984).

The interaction of the disease with other stress factors, such as pesticide residues found in bee-collected pollen and nectar (Kasiotis et al., 2014; Karise et al., 2017; Kiljanek et al., 2017; Böhme et al., 2018; Raimets et al., 2020) may be detrimental to honeybees (Goblirsch, 2018). Several pesticides can affect a bee’s ability to cope with various diseases. For example, simultaneous exposure to glyphosate and difenoconazole, in *Nosema*-infected honeybees, was demonstrated to induce additional physiological stress (Almasri et al., 2021). Furthermore, in an Australian field experiment, combined exposure to thiamethoxam and *N. apis* substantially increased mortality, as well as reduced immunocompetence, in honeybees (Grassl et al., 2018). Overall, pathogen-pesticide interactions, either overlapping or sequential, can reduce honeybee survival and weaken colonies (Alaux et al., 2010; Sánchez-Bayo et al., 2016; Grassl et al., 2018; Almasri et al., 2021). Negative co-effects of *Nosema ceranae* infection and pesticides are previously observed over different life stages of worker honeybees (Glavinic et al., 2019; Tesovnik et al., 2020).

Fast and reliable methods for measuring non-invasive endpoints in living bees are needed. Until now, most studies have focused on the effects of one or multiple stress factors on cellular processes, survival and humoral immune response (Hartfelder et al., 2013; Havard, Laurent, and Chauzat, 2019; Trytek et al., 2022). Other studies focus on behavioural changes, brood development, number of mites or spores counted or estimated (Henry et al., 2017; Bird et al., 2021). However, it is difficult to measure the severity of these interactions on living bees. These methods are usually invasive and the bee will be sacrificed during the experiment. One of the methods allows measurements of living and intact insects might be respirometry, which allows to observe changes in metabolic rate in real-time and in living organisms (Muljar et al., 2012; Mänd and Karise, 2015; Karise et al., 2016; Ploomi et al., 2018; Castro et al., 2021). The traditional survival experiments may hide some important toxicological aspects like the process of recovery from toxicosis (El-Seedi et al., 2022) allowing also repeated measurement of treated individuals.

Nosemosis does not cause rapid death of colonies (Martín-Hernández et al., 2011) and is detectable throughout several seasons (Naudi et al., 2021). However, in combination with other stressors, the severity increases (Straub et al., 2020). The two *Nosema* species differ in their virulence (Martín-Hernández et al., 2011; Naudi et al., 2021), but both affect the bee gut by destroying the epithelial cells (Adl et al., 2005). We hypothesise that this could cause a nutritional deficiency that could be detectable with respirometry measurements. For instance the effects of neurotoxic insecticides are usually easily detectable with respirometry (Kestler, 1991; Muljar et al., 2012; Vinha et al., 2021). We also expect that the combination of the low dose of a neurotoxic insecticide and the pathogen might severely affect the overall metabolic rate of honeybees.

## Materials and methods

### The geographical location and study design

The study was conducted in Estonia in the summer of 2018 in Estonian University of Life Sciences in the insect physiology laboratory of the Chair of Plant Health. The first step was to establish 12 round isolated tents (with diameter of 3 m) covered with insect-proof nets. Each tent accommodated one Italian honeybee (*Apis mellifera ligustica*) colony. All the colonies were equal in size—the nucleus colonies included five frames (three frames of brood, two frames of honey), and the feeder for sugar solution.

**FIGURE 1:**
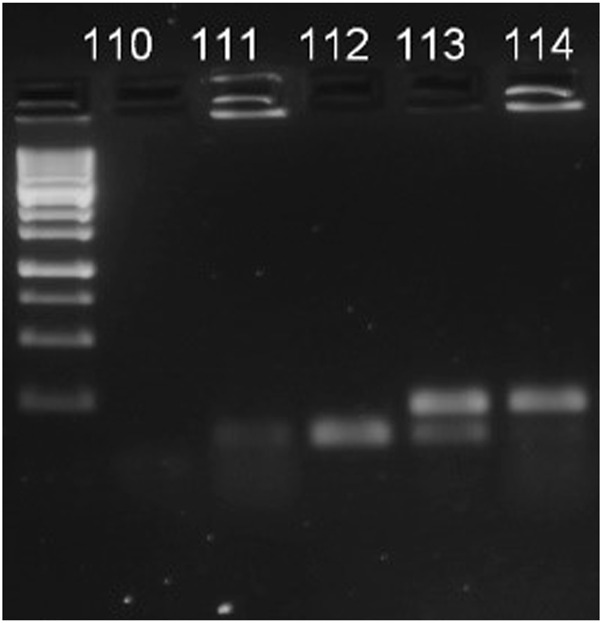
Figure 1 Example of visualisation of M-PCR products. 110 indicates negative control, 111—negative result, 112—N. ceranae, 113—mix of both pathogens, 114—*Nosema apis*.

We repeatedly sampled forager bees from the entrance of the colonies 2 weeks before the experiment to ensure no *Nosema* infection was present. The absence of the *Nosema* spp. was proven by using Multiplex PCR method. The DNeasy Blood and Tissue Kit (Qiagen, Hilden, Germany) was used to isolate DNA, thereafter a Multiplex PCR (M-PCR) was used to identy the species (described in (Naudi et al., 2021)). Following the assay, electrophoretic separation was done to PCR products and visualized in 2% agarose TAE gel (N. apis: band at 321 bp, N. ceranae: band at 218–219 bp, Figure 1).

To obtain the *N. apis* and *N. ceranae* spores, honeybee colonies with inherent clinical nosemosis symptoms were identified by local beekeepers. From these colonies, infected worker bees were collected, midguts with entire hind bodies were removed and crushed using a hand homogenizer in a Bioreba universal separation bag (15 × 28.5 cm), and the spore-bearing suspension was collected. The *Nosema* species were identified by the same Multiplex PCR method. Once the species were identified, centrifugation protocol (Fries et al., 2013a) was used to purify the spores. As the sample purity with this protocol is about 85%, the resulting suspension was cleaned with a 10-micron filter. A flow cytometer (Accuri^®^C6 flow cytometer (Becton Dickinson, BD, Franklin Lakes, NJ, USA) was used to determine the number of spores (described in (Naudi et al., 2021)).

To infect the nosemosis-free colonies, we used three treatments (*N. apis, N. ceranae* and their co-infection) with three replications each. Each treatment consisted of five million spores mixed in 1 L of syrup (mix of 40% fructose, 30% glucose, and 30% sucrose dissolved in heated water). The food was provided weekly for 3 weeks, meaning each honeybee colony received a total of 15 million spores.

According to the calculations, each bee acquired about 1200–1500 spores (colony with 5 frames full of bees—roughly 2000–2500 bees per frame). Two weeks after the final inoculation, mortality and physiology experiments were conducted.

### Pesticide treatment

As additional stressor, the insecticide dimethoate in its pure form (Sigma-Aldrich, 99.6% pure active ingredient) was used. Dimethoate was chosen for this experiment as a positive control. Dimethoate has known to have a negative effect on honeybees with known and established lethal and non-lethal doses (Schuehly, Riessberger-Gallé, and Hernández López, 2021).

Dimethoate was mixed into sugar syrup (water and sugar 1:1). 20 μg of dimethoate (far below the LD50 to honeybees) was added per 1 L of syrup. The pesticide concentration was chosen according to the survey conducted in Poland where 20 µg of dimethoate per 1 kg of honey was found. (Bargańska, Ślebioda, and Namieśnik, 2013). Both in control and treatment groups, the bees were fed *ad libitum*. The colonies were monitored daily and empty feeders were replaced with full ones. We did not monitor the exact amount of feed consumed by individual bees. However, based on standard calculations about honeybee daily food intake (Rodney and Purdy, 2020), we calculated the approximate amount of food consumed and thus possible dimethoate intake. The bees received 20 µg of dimethoate per litre through feed syrup. Considering that one worker bee consumes 22 mg of sugar syrup per day, maximum of 0.0003 µg per day of dimethoate was consumed by one bee.

### Mortality assessment

To assess the effects of dimethoate and nosemosis on honeybee mortality, we created special micro-colonies in small wooden cages (9 × 7 × 4 cm), with plastic mesh sides. 20 young worker bees, taken from each nucleus colony described above, were placed in each cage. The cages were kept in climate chamber (controlled temperature, humidity and total darkness, Sanyo MLR-351H, Osaka, Japan), where ambient temperature was 34.5 °C and the relative humidity was 60% to mimic the normal conditions of a bee colony. Cages were equipped with two 1.5 ml Eppendorf tubes (one containing feed syrup (1:1 sugar-water) and the other containing distilled water), where the bees had free access. We created two sets of cages per each hive—one to test the effect of the pathogen, the other to test the effect of adding chronical exposure to a non-lethal dose of dimethoate. From each colony, we created three cages, while we had three colonies in every treatment group, which makes 9 cages per treatment group. Treatments were distributed as follows: control, control + dimethoate, *N. apis*, *N. apis* + dimethoate, *N. ceranae*, *N. ceranae* + dimethoate, *Nosema* co-infection, *Nosema* co-infection + dimethoate. The observation lasted 25 days, during which dead bees were counted every 24 h.

### Respiratory measurements

Separate cages with honeybees needed for respiratory measurements were prepared additionally to those used in mortality assessments. 20 bees were collected from each hive, and acclimated for 12 h before the measurements started. Measurements of the 10 individuals lasted about 10 h, thus all the honeybees from all treatments had been exposed to the dimethoate 12–21 h, and this scheme was followed for each treatment.

The bee respiration rate was determined, using flow-through respirometer LI-7000 (LiCor, Lincoln, Nebraska, USA). Measurements were performed at room temperature (22°C ± 1°C). Each bee spent 50 min in respirometer chamber. The air circulating in the analyzer was taken from the laboratory environment and was passed through magnesium perchlorate Mg(ClO_4_)_2_ and sodium hydroxide NaOH to remove water vapor and CO_2_. The volume of air passing through the system in each minute was 600 ml. The measurement of the metabolic rate of a single bee lasted 60 min, which included including a 5-min machine calibration time to achieve 0-level and 5 min for calming down from handling stress of honeybees—both which were excluded from the analysis. Thus the metabolic rate was calculated based on 50 min measurement. During this time, the machine reached its 0-level registration stage.

### Statistical analysis

The statistical software Statistica (Dell^TM^ Statistica^TM^, StatSoft) was used to process and analyze the data of the experiment. The respiratory data was normalized by log-transforming (using logarithmic values of) the data. The effects of the variables ‘Nosema treatment’ and ‘dimethoate treatment’ were analyzed using Main-effect ANOVA followed by Fisher LSD *post hoc* test. For mortality data we used the General Linear Models Repeated Measures Analyses of Variance using the ‘Time’ as within-effects factor and ‘Nosema treatment’ and ‘dimethoate treatment’ as categorical factors. To understand when the different factors started to affect the honeybee longevity, we allowed the model to calculate Univariate Results for each day.

## Results

### Effect on honeybees metabolism

By the end of the experiment samples were collected and analysed. The results showed 4872–65940 spores per bee throughout all treatments. In average, *N. apis* 9417 ± 4544,5, *N. ceranae* 42966 ± 22974 and Nosema mix 7434 ± 2702 spores.

**FIGURE 2:**
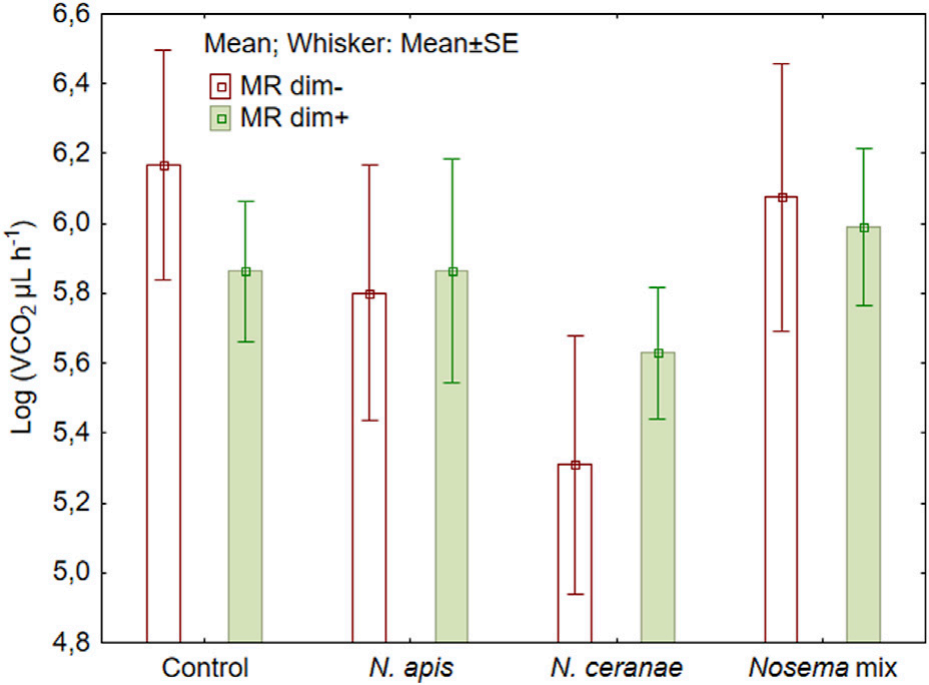
Mean metabolic rate (MR) of honeybees (N = 10) during 50 min of measurements after overlapping exposure to *Nosema apis*, *N. ceranae* or mix of both with or without a sublethal dose of dimethoate (dim−/dim+).

We did not detect statistically significant changes in metabolic rate of honeybees neither by the *Nosema* treatment (Figure 2) (F (3, 72) = 0.85, *p* = 0.47) nor by adding dimethoate at sublethal level (F (1, 72) = 0.80, *p* = 0.37) (Figure 2). The variations within the treatments were relatively large, ranging from 61.8 µL CO_2_/L of air to 3456.6 µL CO_2_/L. The lowest result was recorded in honeybees infected with *N. ceranae* spores (the difference from the control was nearly significant, Fisher LSD test: *p* = 0.057) and the highest result in those bees infected with *N. apis* spores that also had received dimethoate in the feed.

### Mortality

**FIGURE 3:**
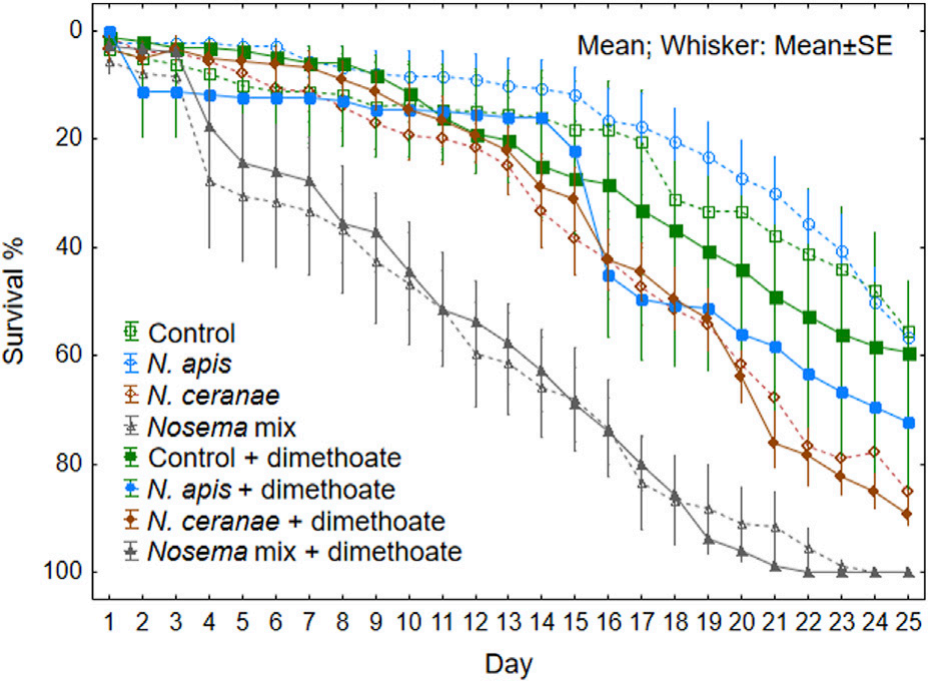
Survival rate of honeybees (N = 9 cages, 20 honeybees in each) per treatment group during 25 days of observation after overlapping sposure to *Nosema apis*, *N. ceranae* or mix of both with or without a sublethal dose of dimethoate chronically in the feed.

Mortality rate in *Nosema* infected bees increased significantly on the day 4 of the experiment (F (3, 64) = 17.47, *p* < 0.001) (see Univariate Results for each day in Supplementary Material Supplementary Table S1). Additional stress caused by dimethoate was generally not significant (F (1, 64) = 0.87, *p* = 0.35). However we observed significant effect on bee mortality on day 21 in the *N. apis* treatment group (Figure 3; Supplementary Table S1).

Mortality rate was lowest in the *N. apis* treatment group during the full extent of the experiment, being even slightly slower than that of the control groups (Figure 3). In the *N. ceranae* treatment groups, the mortality rate was initially slow similar to the control and *N. apis* groups, but increased rapidly after 2 weeks from the start of the experiment. The combination of both *Nosema* spp. resulted in higher mortality rate single pathogens and the difference was obvious already after few days followed by constant increase in mortality rate.

## Discussion

Our results suggest that *N. apis* as a stressor does not significantly affect the respiration rate on individual level in honeybees. Still, the *N. ceranae* had a greater impact compared to *N. apis*. Different studies also found the same difference between the two pathogens (Higes et al., 2010; Goblirsch, 2018; Sinpoo et al., 2018). *N. ceranae* is described as more energy demanding on the host, indicated by higher feed consumption (Martín-Hernández et al., 2011).

Dimethoate has been demonstrated to be lethal to honeybees when administered at concentrations of 0.2 µg per bee (Baskar, Sudha, and Tamilselvan, 2016). Sublethal concentration of dimethoate may increase the bumblebee’s food consumption (Cornement et al., 2017), it is possible that similar increase took place in our study. However, as the feed consumption was not monitored, the consumed amount of feed is based on average nutritional needs of 0.0003 µg per day.

Different stressors can result in different responses in honeybees, including altered metabolism (Even, Devaud, and Barron, 2012). In the present study, the absence of any significant effect may have resulted from high variability within the data accompanied with relatively low spore loads (Figure 2.). Such variability can be due to the individual physiological characteristics of bees, as each individual has a different ability to withstand stress. In addition to the treatments, further stress is induced by handling the bees when putting them in the analyser and limiting their movement. To reduce the impact of handling stress, it could be helpful to extend the period of analysis, during which the individuals could get used to new and more unpleasant conditions (Mänd et al., 2005). In the case of acclimated bees, the variability in the dataset may somewhat decrease (Even, Devaud, and Barron, 2012). However, based on our experiences, honeybees do not tolerate long detainment in the analyser.

Individual stress factors (*N. apis, N. ceranae* and dimethoate) had no individual impact on honeybee mortality. The authors suggest that this was because the bees used in the experiments were mainly adult bees. The adult honeybees can be more resistant to stress induced by pathogens (Roberts and Hughes, 2014). The selective breeding throughout the years has also resulted in more tolerant bees that are more capable to maintain close to normal functionality even when infected with *Nosema spp* (Kurze et al., 2016).

Spore counts have often been used to describe the severity of the disease (Botías et al., 2013; Vidau et al., 2014; Kurze et al., 2016). It is proven that 10–33 000 spores per bee is sufficient to induce infection (Fries et al., 2013). In an experiment carried out in Sweden, where mixture of *N. apis* and *N. ceranae* spores were used. There the number of spores used to infect honey bees ranged from 10 to 10,000 per bee. Regardless of the mixed pathogen spore ratio used, the number of spores equalized by day 12 of the experiment and infection occurred in both cases (Forsgren and Fries, 2010). From the collected and analyzed bees, after the experiment, we conclude that the amount of spores used in this work were sufficient to induce infection, cause stress and alter the metabolic rate. Natural resistance to nosemosis of the bees used in our experiment is unknown. It is demonstrated that in artificial laboratory conditions can result in a longer lifespan of bees (Human et al., 2013). In this regard, the physical capacity to tolerate stress in laboratory conditions can be greater than in the field conditions.

The combined effect of multiple stressors, in the form of *N. apis* and *N. ceranae*, resulted in a significantly higher stress response, expressed by higher honeybee mortality. Control groups, both with and without dimethoate, and also infection with solely *N. apis*, resulted in 50% of the specimens still alive at the end of the experiment (25 days). Approximately half of the bees (53%) were alive on day 20. This is similar to the results of Vidau (2011), where specimens were infected only with *N. ceranae,* but the additionally exposure to fipronil and thiacloprid caused the mortality of 82% and 71% respectively (Vidau et al., 2011). In our study, *N. ceranae* and the overlapping exposure to dimethoate, resulted both in the 60% mortality of individuals on the day 20 of observation (Figure 2). But with *N. apis*, our results clearly demonstrate the additive stress from the dimethoate—already at Day15, the mortality increased rapidly being similar to the mortality level honeybees of *N. ceranae* treatment groups. Given that the 24 h LD_50_ of dimethoate for honey bee workers is 0.126 µg/bee, dimethoate alone in the concentrations chosen in our experiment, is unlikely to cause significant mortality.

The maximum amount of dimethoate that one bee can take per day in this experiment remained significantly lower, as mentioned above. The negative interaction of different pesticides and nosemosis has been proven in various researches. The interaction is often manifested in the fact that a sublethal dose of pesticide causes faster growth of *Nosema* spores in the nosemosis honeybees (Alaux et al., 2010; Vidau et al., 2011; Aufauvre et al., 2012; Pettis et al., 2012). According to the present study, the combined effects of stressors results in shorter lifespan of worker bees even at very low doses, which hardly impact the test results at shorter time scale as shown by our respirometry experiment. Therefore it is important to pay attention to the honeybee colony health. Beekeepers should be aware about combined effects of stressors and try to avoid adding any synthetic pesticides as parasite or disease treatment. Even if it would be hard to avoid agricultural pesticides, avoiding synthetic veterinary medicines in beehives, is something, every beekeeper can do to protect their honeybees.

## Data Availability

The raw data supporting the conclusions of this article will be made available by the authors, without undue reservation.
