# Supplementary material for: Overlapping exposure effects of pathogen and dimethoate on honeybee (Apis mellifera Linnaeus) metabolic rate and longevity
Source: Front Physiol. 2023 Jun 6;14:1198070. doi: 10.3389/fphys.2023.1198070 (PMC10279948; doi:10.3389/fphys.2023.1198070)
Supplement: Supplementary file 1 [file Table1.DOCX]

Supplementary materials

Table S1. Mortality experiment significant results are marked with red colour. General linear model repeated measures ANOVA.

|  | Nosema treatment | Dimethoate | Nosema treatment*Dimethoate |
| --- | --- | --- | --- |
| Day 1 F | 1.47 | 1.43 | 1.24 |
| Day 1 P | 0.2299 | 0.2366 | 0.3028 |
| Day 2 F | 0.29 | 0.07 | 1.42 |
| Day 2 P | 0.8309 | 0.7904 | 0.2443 |
| Day 3 F | 0.31 | 0.01 | 1.43 |
| Day 3 P | 0.8180 | 0.9296 | 0.2420 |
| Day 4 F | 3.78 | 0.11 | 0.89 |
| Day 4 P | 0.0147 | 0.7416 | 0.4518 |
| Day 5 F | 4.90 | 0.07 | 0.64 |
| Day 5 P | 0.0039 | 0.7789 | 0.5918 |
| Day 6 F | 4.96 | 0.13 | 0.63 |
| Day 6 P | 0.0037 | 0.7157 | 0.5971 |
| Day 7 F | 5.36 | 0.21 | 0.39 |
| Day 7 P | 0.0023 | 0.6471 | 0.7581 |
| Day 8 F | 7.77 | 0.10 | 0.34 |
| Day 8 P | 0.0002 | 0.7556 | 0.7989 |
| Day 9 F | 7.90 | 0.27 | 0.39 |
| Day 9 P | 0.0001 | 0.6042 | 0.7621 |
| Day 10 F | 10.44 | 0.029 | 0.20 |
| Day 10 P | <0.001 | 0.8634 | 0.8928 |
| Day 11 F | 13.33 | 0.05 | 0.15 |
| Day 11 P | <0.001 | 0.8260 | 0.9292 |
| Day 12 F | 16.41 | 0.01 | 0.31 |
| Day 12 P | <0.001 | 0.9166 | 0.8204 |
| Day 13 F | 17.24 | 0.05 | 0.24 |
| Day 13 P | <0.001 | 0.8323 | 0.8663 |
| Day 14 F | 17.08 | 0.10 | 0.35 |
| Day 14 P | <0.001 | 0.7531 | 0.7893 |
| Day 15 F | 16.62 | 0.33 | 0.52 |
| Day 15 P | <0.001 | 0.5674 | 0.6705 |
| Day 16 F | 13.45 | 2.57 | 1.20 |
| Day 16 P | <0.001  0 | 0.1136 | 0.3178 |
| Day 17 F | 16.25 | 2.46 | 1.85 |
| Day 17 P | <0.001 | 0.1220 | 0.1472 |
| Day 18 F | 14.61 | 1.63 | 1.40 |
| Day 18 P | <0.001 | 0.2070 | 0.2499 |
| Day 19 F | 16.00 | 2.40 | 0.96 |
| Day 19 P | <0.001 | 0.1265 | 0.4175 |
| Day 20 F | 17.13 | 3.63 | 0.96 |
| Day 20 P | <0.001 | 0.0613 | 0.4177 |
| Day 21 F | 17.15 | 5.25 | 0.67 |
| Day 21 P | <0.001 | 0.0252 | 0.5728 |
| Day 22 F | 15.73 | 3.47 | 0.92 |
| Day 22 P | <0.001 | 0.0672 | 0.4380 |
| Day 23 F | 15.35 | 3.20 | 0.90 |
| Day 23 P | <0.001 | 0.0782 | 0.4457 |
| Day 24 F | 14.64 | 2.77 | 0.52 |
| Day 24 P | <0.001 | 0.1008 | 0.6694 |
| Day 25 F | 13.62 | 1.24 | 0.39 |
| Day 25 P | <0.001 | 0.2688 | 0.7605 |
